# A tailored approach to cardioneuroablation for reflex syncope and functional bradycardia: results from the ELEGANCE multicentre study

**DOI:** 10.1093/europace/euaf320

**Published:** 2025-12-30

**Authors:** Carlo Gigante, Diego Penela, Daniel Viveros, Giulio Falasconi, Lucio Teresi, Alessia Chiara Latini, David Soto-Iglesias, Paula Franco-Ocaña, Pietro Francia, José Alderete, Dario Turturiello, Aldo Francisco Bellido, Fatima Zaraket, Chiara Valeriano, Roberta Mea, Bruno Tonello, Lautaro Sanchez-Mollá, Carmine De Lucia, Maria Matiello, Juan Fernández-Armenta, Rodolfo San Antonio, Andrea Saglietto, José-Tomás Ortiz-Pérez, Julio Martí-Almor, Antonio Berruezo

**Affiliations:** Arrhythmia Department, Heart Institute, Teknon Medical Center, c. Villana, 12, Barcelona 08022, Spain; Facultat de Medicina I Ciències de la Salut, Universitat de Barcelona (UB), c. Casanova, 143, Barcelona 08036, Spain; Arrhythmia Department, Humanitas Research Hospital IRCCS, Rozzano, Milan, Italy; Arrhythmia Department, Heart Institute, Teknon Medical Center, c. Villana, 12, Barcelona 08022, Spain; Facultat de Medicina I Ciències de la Salut, Universitat de Barcelona (UB), c. Casanova, 143, Barcelona 08036, Spain; Arrhythmia Department, Heart Institute, Teknon Medical Center, c. Villana, 12, Barcelona 08022, Spain; Facultat de Medicina I Ciències de la Salut, Universitat de Barcelona (UB), c. Casanova, 143, Barcelona 08036, Spain; Arrhythmia Department, Heart Institute, Teknon Medical Center, c. Villana, 12, Barcelona 08022, Spain; Facultat de Medicina I Ciències de la Salut, Universitat de Barcelona (UB), c. Casanova, 143, Barcelona 08036, Spain; Arrhythmia Department, Humanitas Research Hospital IRCCS, Rozzano, Milan, Italy; Department of Biomedical Sciences, Humanitas University, Pieve Emanuele-Milan, Italy; Arrhythmia Department, Heart Institute, Teknon Medical Center, c. Villana, 12, Barcelona 08022, Spain; Arrhythmia Department, Heart Institute, Teknon Medical Center, c. Villana, 12, Barcelona 08022, Spain; Cardiology Department, St. Andrea Hospital, Rome, Italy; Department of Clinical and Molecular Medicine, Sapienza University, Rome, Italy; Arrhythmia Department, Heart Institute, Teknon Medical Center, c. Villana, 12, Barcelona 08022, Spain; Facultat de Medicina I Ciències de la Salut, Universitat de Barcelona (UB), c. Casanova, 143, Barcelona 08036, Spain; Arrhythmia Department, Heart Institute, Teknon Medical Center, c. Villana, 12, Barcelona 08022, Spain; Facultat de Medicina I Ciències de la Salut, Universitat de Barcelona (UB), c. Casanova, 143, Barcelona 08036, Spain; Arrhythmia Department, Heart Institute, Teknon Medical Center, c. Villana, 12, Barcelona 08022, Spain; Arrhythmia Department, Hospital El Pilar, Barcelona, Spain; Arrhythmia Department, Heart Institute, Teknon Medical Center, c. Villana, 12, Barcelona 08022, Spain; Arrhythmia Department, Humanitas Research Hospital IRCCS, Rozzano, Milan, Italy; Arrhythmia Department, Heart Institute, Teknon Medical Center, c. Villana, 12, Barcelona 08022, Spain; Department of Biomedical and Clinical Sciences, University of Milan, Milan, Italy; Arrhythmia Department, Heart Institute, Teknon Medical Center, c. Villana, 12, Barcelona 08022, Spain; Arrhythmia Department, Heart Institute, Teknon Medical Center, c. Villana, 12, Barcelona 08022, Spain; Arrhythmia Department, Heart Institute, Teknon Medical Center, c. Villana, 12, Barcelona 08022, Spain; Arrhythmia Department, Hospital General de Catalunya, Barcelona, Spain; Arrhythmia Department, Puerta del Mar University Hospital, Cádiz, Spain; Cardiology Department, Bellvitge University Hospital, BIO-HEART Cardiovascular Research Group (IDIBELL), l'Hospitalet de Llobregat, Barcelona, Spain; Division of Cardiology, Cardiovascular and Thoracic Department, Città Della Salute e Della Scienza Hospital, Turin, Italy; Department of Medical Sciences, University of Turin, Turin, Italy; Arrhythmia Department, Heart Institute, Teknon Medical Center, c. Villana, 12, Barcelona 08022, Spain; Facultat de Medicina I Ciències de la Salut, Universitat de Barcelona (UB), c. Casanova, 143, Barcelona 08036, Spain; Arrhythmia Department, Heart Institute, Teknon Medical Center, c. Villana, 12, Barcelona 08022, Spain; Arrhythmia Department, Heart Institute, Teknon Medical Center, c. Villana, 12, Barcelona 08022, Spain

**Keywords:** Cardioneuroablation, Reflex syncope, Vasovagal syncope, Carotid sinus syndrome, Bradycardia, Ganglionated plexi, Head-up tilt test, Tailored approach

## Abstract

**Aims:**

Cardioneuroablation (CNA) is a catheter-based intervention for reflex syncope and functional bradyarrhythmias that consists in the modulation of the parasympathetic cardiac autonomic nervous by targeting ganglionated plexi (GPs).

To compare an ablation strategy of selective GP targeting based on clinical phenotype (tailored approach) vs. the standard approach of targeting all GPs (standard approach).

**Methods and results:**

This is a prospective, multicentre European study (ELEGANCE study), including 123 patients who underwent CNA (73 men; median age 50 years). Among them 54 (44%) were treated with a tailored approach, targeting the superior paraseptal ganglionated plexus (SPSGP) for sinus node dysfunction and the inferior paraseptal ganglionated plexus (IPSGP) for AV block. Procedural data and clinical outcomes were compared with the remaining 69 patients treated using a standard approach.

Clinical phenotypes included isolated functional sinus node dysfunction (43.1%), isolated functional AV block (9.8%), and dual presentations (47.2%). In the tailored group 1.6 ± 0.7 GPs were targeted per patient. Compared to the standard approach, the tailored group had significantly shorter procedure times (63 vs. 85 min, *P* = 0.005) and reduced RF time (5.4 vs. 10.4 min, *P* < 0.001). Acute procedural success (tailored: 93% vs. standard: 90%, *P* = 0.98) and the increase in heart rate (tailored: 40 ± 30.7% vs. standard: 40 ± 31.4%, *P* = 0.96) were similar between groups. During a median 15.9 months [IQR: 9.8, 24.6] follow-up, there were no differences in syncope recurrence rate (log-rank *P* = 0.96). Inappropriate sinus tachycardia occurred in 8.1% of patients, (tailored 8.6% vs. standard 7.4%; *P* = 0.79).

**Conclusion:**

An individualized CNA strategy, simplified by targeting specific GPs according to patient’s pathophysiology, achieved outcomes equivalent to the standard approach while improving procedural efficiency through reduced RF delivery, shorter procedure duration, and limited ablation extent.

## Graphical Abstract

**Graphical Abstract:**
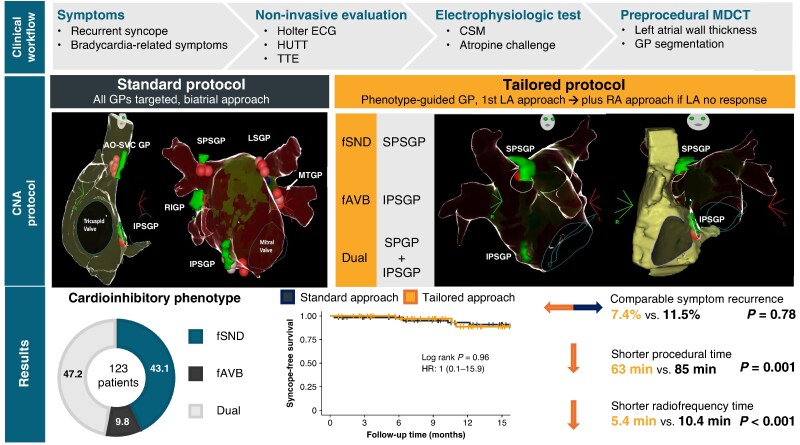
Patients with recurrent syncope or bradyarrhythmia symptoms underwent non-invasive evaluation (Holter ECG, HUTT, TTE), electrophysiologic testing (CSM, atropine), and MDCT for LA wall thickness and GP segmentation. Standard CNA targeted all GPs biatrially, while the tailored protocol ablated SPSGP for fSND and IPSGP for fAVB, with RA ablation if no LA response. Tailored CNA achieved similar outcomes with shorter procedure and RF times. CNA, cardioneuroablation; CSM, carotid sinus massage; ECG, electrocardiogram; fAVB, functional atrioventricular block; fSND, functional sinus node dysfunction; GP, ganglionated plexus; GPs, ganglionated plexi; HUTT, head-up tilt test; IPSGP, inferior paraseptal ganglionated plexus; LA, left atrium; MDCT, multidetector computed tomography; RA, right atrium; RF, radiofrequency; SPSGP, superior paraseptal ganglionated plexus; TTE, transthoracic echocardiography.

What’s new?

- This study introduces a phenotype-guided, tailored cardioneuroablation (CNA) strategy that selectively targets specific ganglionated plexi according to each patient’s predominant clinical presentation, primarily targeting the superior paraseptal ganglionated plexus for sinus node dysfunction and the inferior paraseptal ganglionated plexus for atrioventricular block symptoms.
- It represents the first prospective, multicentre comparison between a tailored CNA approach and the conventional comprehensive ‘all-plexi’ ablation strategy.
- The tailored protocol achieved comparable clinical efficacy and syncope-free survival, while significantly reducing procedural duration (63 vs. 85 min) and radiofrequency delivery time (5.4 vs. 10.4 min).
- Selective targeting reduced the extent of ablation and potential tissue injury without compromising acute procedural success or safety.
- These findings provide proof of concept for a simplified and individualized CNA approach, supporting its integration into contemporary clinical practice.

## Introduction

Cardioneuroablation (CNA) has emerged as a promising alternative to conventional therapies in selected cases of vasovagal cardioinhibitory reflex syncope and functional bradyarrhythmias.^1, 2^ This innovative approach targets the heart's intrinsic autonomic nervous system, specifically the ganglionated plexi (GPs), which are clusters of autonomic nerve bodies embedded in the epicardial surface of the heart.^3^ Through an intricate network of neural connections, they modulate both sinoatrial node function and atrioventricular node conduction.

The technique, first introduced two decades ago, has gained increasing attention and implementation over the past years, offering a therapeutic approach that can either postpone or eliminate the need for permanent pacemaker implantation in appropriately selected patients.^4^

Observational studies and recent randomized controlled trials have demonstrated encouraging results regarding CNA's efficacy and safety.^5,6^

However, several methodological and clinical considerations remain unresolved.^7^ Relevant questions persist regarding the optimal approach to GP detection, the procedure approach (i.e. right atrium [RA] vs. biatrial), and the ablation sequence.^8^

Up to six GPs have been identified as potential targets during ablation procedures^9^; however, not all GPs have demonstrated the same therapeutic effect. The superior paraseptal ganglionated plexus (SPSGP) is the final common pathway for the right vagus nerve innervating the sinoatrial node, and its ablation often results in sinus rhythm heart rate increase. In contrast, the inferior paraseptal ganglionated plexus (IPSGP) serves as the final common pathway for the left vagus nerve innervating the AV node, and its ablation is critical in cases of vagally induced AV block.^7^ The distinct innervations of each GP group provide an opportunity for personalized CNA tailored to the predominant clinical bradyarrhythmia phenotype. This approach has the potential to reduce procedural complexity, radiofrequency (RF) delivery, and associated complications. However, so far, the most common approach involves unselective ablation of all GPs, regardless of the patient’s clinical presentation.^7, 10^

This study aims to describe the results of a tailored approach for an ablation strategy of selective GP targeting based on clinical phenotype, and to compare procedural data and clinical outcomes with the standard approach of targeting all GPs.

## Methods

### Study population

The ELEGANCE is a prospective multicentre study that includes consecutive patients who underwent CNA in 5 centres.^11^ As part of a previously published protocol, all patients undergo a comprehensive evaluation before CNA including 24-h Holter monitoring, head-up tilt testing (HUTT), and complete electrophysiology studies with carotid sinus massage and atropine testing.

Of 123 patients included in the study, 54 (44%) were treated with a tailored approach (tailored group). In the remaining 69 (56%) patients, all GPs were targeted for ablation (standard group). We are reporting the analysis of a prospectively collected data comparing both approaches that were sequentially implemented, first the standard approach and thereafter the tailored approach, with all patients included consecutively. The study received approval from our institutional Ethics Committee and all participants provided written informed consent.

Patients’ phenotypes were defined based on the predominant clinical and electrophysiological manifestations. Three distinct phenotypes were identified: (i) Isolated functional sinus node dysfunction (fSND) characterized by symptomatic bradycardia, sinus pauses, or chronotropic incompetence without significant AV conduction abnormalities; (ii) isolated functional AV block (fAVB) presenting with second-degree, or high-degree fAVB as the predominant manifestation; (iii) dual phenotype exhibiting both sinus node dysfunction and AV conduction disturbances. Phenotype classification was based on baseline electrocardiographic findings, 24-h or subcutaneous Holter monitoring, HUTT, electrophysiological study parameters, and clinical presentation patterns.

### Cardioneuroablation procedure

All CNA procedures were guided by imaging, as previously published.^3^ For this purpose, all patients underwent a preprocedural cardiac multidetector computed tomography (MDCT). The images were acquired during an inspiratory breath-hold using retrospective ECG-gating technique with tube current modulation set between 50% and 100% of the cardiac cycle. Three-dimensional anatomical bi-atrial maps were generated using ADAS 3D™ software (Adas3D Medical SL, Barcelona, Spain). Epicardial fat pads near anticipated GP locations were identified using attenuation values between −190 and −30 Hounsfield units (HU). CNA procedures were conducted under general anaesthesia and utilizing HFLTV ventilation protocol.^12^ Transseptal puncture was guided by transoesophageal echocardiography. All procedures were performed with single catheter technique.^13^ Using either CARTO 3 or NavX systems, a fast-anatomical map (FAM) of the entire left atrium (LA) anatomy and pulmonary veins (PVs) was acquired and subsequently integrated with the post-processed CT derived 3D maps, that include both epicardial fat pads localization and LA wall thickness (LAWT) information. Ablation was performed point-by-point using an irrigated-tip, contact force–sensing catheter. For all applications, RF power was limited to 45 W, and RF delivery was tailored based on the local left atrial wall thickness, as shown in Supplementary material online, *Table S1*.

### Treatment strategy

All procedures were performed using a biatrial approach, enabling access to both left- and right-sided GP sites. In the Standard Group, ablation systematically targeted all major GPs: the left superior GP (LSGP) between the left superior pulmonary vein and LA appendage, the Marshall tract GP (MTGP) in the left PV carina, the left inferior GP (LIGP) posterior to the left inferior PV, the IPSGP between the LA posterior wall and coronary sinus, the SPSGP between the right superior PV and superior vena cava, the right inferior GP (RIGP) between the right PVs, and the aorta-superior vena cava GP (Ao-SVC GP). Both SPSGP and IPSGP were approached from left and RA aspects, with combined responses to RF delivery recorded.

In the Tailored Group, a strategy targeting specific GPs based on the patient’s underlying pathophysiology was implemented, initially restricted to the left atrium. Acute success (see below for definitions) was reassessed after ablation of each GP to determine whether RAl ablation or additional GP targets were required.

Patients presenting with sinus node dysfunction underwent ablation of the SPSGP, starting from the left atrium and, if necessary, proceeding to the right SPSGP and then the Ao–SVC GPs. If acute success was not achieved, remaining GPs could be targeted at the operator’s discretion. For those with isolated atrioventricular block, ablation was initially limited to the left IPSGP, with additional targeting from the right side-only in the absence of acute success.

In patients with combined sinus node dysfunction and AV block, both SPSGP and IPSGP were considered primary targets.

### Procedural endpoints

Acute procedural success was defined according to the patient’s phenotype. In cases of fSND, successful CNA was determined by a post-ablation increase in heart rate greater than 25% from baseline, accompanied by a negative response to post-procedural carotid sinus massage and less than 25% heart rate increase response to atropine administration at the end of the procedure. In the subgroup of fSND patients presenting with persistent functional bradycardia, an additional requirement was achievement of at least 70% of the heart rate response to atropine previously documented during the baseline electrophysiological evaluation. In patients, fAVB phenotype, procedural success was defined by the resolution of AV block and/or a reduction in the PR interval of at least 25% from baseline, combined with a negative response to post-procedural carotid sinus massage and the absence of further PR shortening following atropine administration. In patients with a dualphenotype, both criteria were required. Extracardiac vagal stimulation (ECVS), when available, was used to confirm acute success at the end of the procedure, with complete vagal denervation—defined as the persistent absence of any cardioinhibitory response to ECSV reproducing the specific clinical phenotype. See Supplementary material online, material for ECVS methodology.

### Follow-up and endpoints

Follow-up included scheduled clinical evaluations, 12-lead ECGs and Holter monitoring at 1-, 3-, 6-, and 12-month post-procedure. A HUTT was performed at 3 months. The primary endpoint of the study was the spontaneous recurrence of symptoms that had initially prompted referral for CNA, including syncope or pre-syncope episodes.

### Statistical analysis

Continuous variables were expressed as mean ± standard deviation for normally distributed data, or median with interquartile range (IQR) for non-normally distributed data. Categorical variables were presented as absolute numbers and percentages. For within-group comparisons of pre- and post-ablation continuous variables, paired *t*-tests were employed. Between-group comparisons of proportions were conducted using chi-square tests, or Fisher's exact test when appropriate. Kaplan–Meier survival analysis was utilized to assess syncope-free survival over the follow-up period. Differences in survival between the standard and tailored approach groups were compared using the log-rank test. Statistical significance was set at *P* < 0.05. All analyses were performed using R version 4.2.0 (R Foundation for Statistical Computing, Vienna, Austria)

## Results

### Baseline characteristics

**Figure 1:**
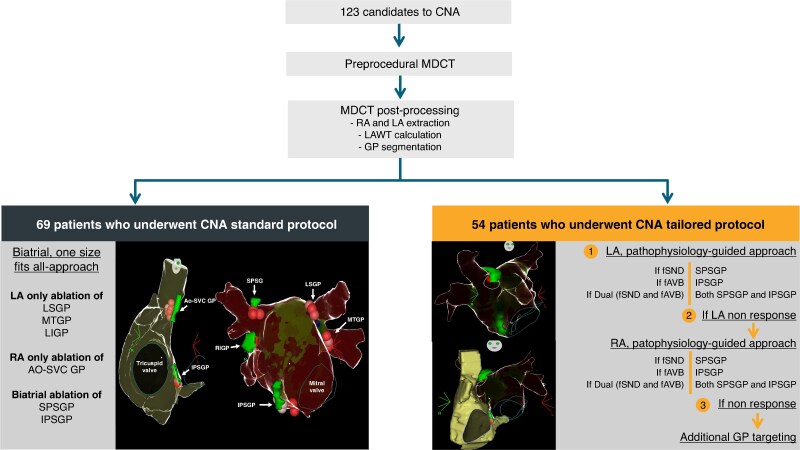
Study flowchart. Study flowchart and CNA strategies. A total of 123 patients underwent preprocedural MDCT with LA and RA extraction, LA wall thickness measurement, and GP segmentation. Sixty-nine patients were treated with the standard protocol, consisting of a biatrial, non-selective ablation of all GPs (LSGP, MTGP, LIGP, AO-SVC GP, SPSGP, and IPSGP). Fifty-four patients were treated with a tailored protocol, beginning with a pathophysiology-guided LA approach (SPSGP for fSND, IPSGP for fAVB, or both if dual), followed by RA ablation if no LA response, and additional GP targeting if still non-responsive. AO-SVC GP, aorto–superior vena cava ganglionated plexus; CNA, cardioneuroablation; fAVB, functional atrioventricular block; fSND, functional sinus node dysfunction; GP, ganglionated plexus; IPSGP, inferior paraseptal ganglionated plexus; LA, left atrium; LAWT, left atrial wall thickness; LIGP, left inferior ganglionated plexus; LSGP, left superior ganglionated plexus; MDCT, multidetector computed tomography; MTGP, mitral ganglionated plexus; RA, right atrium; SPSGP, superior paraseptal ganglionated plexus.

From February 2021 to April 2025, a total of 123 patients underwent CNA [73 men; median age: 52.0 (IQR: 40.0, 61.0) years], as part of the ELEGANCE multicentre study (*Figure 1*). Among these, three patients had structural heart disease (two ischemic and one hypertensive), while the remaining had no structural cardiac abnormalities. *Table 1* shows the baseline characteristics in the entire population depending on the CNA approach (tailored vs. standard).

**Table 1:** Characteristics of study patients according to treatment group

	Standard (*n* = 69)	Tailored (*n* = 54)	*P*-value	Total (*n* = 123)
Female	25 (37.3)	25 (47.2)	0.37	50 (41.7)
Age, years	56 [43.5, 63]	47 [34, 58]	0.07	50 [36, 63]
Number of syncopes	3 [0, 5]	5 [0, 10]	0.10	3 [0, 6]
Holter minimum HR, bpm	42 [36, 50]	42.5 [35, 51]	0.61	42 [35, 50]
Holter mean HR, bpm	64 [55.5, 71.5]	66 [54, 75.5]	0.62	65 [55, 73]
Holter maximum HR, bpm	109 [95, 121]	119 [96.5, 128]	0.34	110 [95, 126.3]
Hypertension	11 (16.7)	8 (15.1)	1.00	19 (15.4)
Dyslipidaemia	8 (12.1)	4 (7.5)	0.60	12 (9.8)
Diabetes mellitus	1 (1.5)	0 (0.0)	1.00	1 (0.8)
Smoker	1 (1.5)	4 (7.7)	0.23	5 (4.1)
Sleep apnoea	3 (4.5)	1 (1.9)	0.77	4 (3.3)
Atrial fibrillation	11 (16.7)	2 (3.7)	0.19	13 (10.6)
***EP study***				
AH interval, ms	90 [74, 114.5]	90 [69, 107]	0.58	90 [72, 110]
HV interval, ms	51 [46, 55.7]	48 [46.75, 52.25]	0.22	50 [46, 54.7]
PW interval, ms	450 [365, 565]	430 [350, 610]	0.72	450 [360, 617.5]
CSNRT, ms	255.50 [150, 344.5]	260 [210, 450]	0.46	260 [200, 390]
Atropine HR increase, %HR	72 [52.6, 85.3]	82 [66.5, 95.5]	0.12	72.14 [53.2, 91.5]
**Head-up tilt test**				
Negative	4 (6.0%)	3 (5.9%)	0.22	7 (5.7%)
Vasodepressor (type 3)	2 (3.0%)	5 (9.8%)		7 (5.7%)
Mixed (type 1)	11 (16.4%)	3 (5.9%)		14 (11.4%)
Cardio-inhibitory type 2A	12 (17.9%)	6 (11.8%)		18 (14.6%)
Cardio-inhibitory type 2B	23 (34.3%)	23 (45.1%)		46 (37.4%)
**Clinical vagal phenotype**				
fSND	31 (44.9%)	22 (40.7%)	0.64	53 (43.1%)
fAVB	8 (11.6%)	4 (7.4%)	0.43	12 (9.8%)
Dual	30 (43.5%)	28 (51.9%)	0.35	58 (47.2%)

Data are presented as mean ± standard deviation, median [interquartile range], or *n* (%).

AH, atrium-His interval; CNA, cardioneuroablation; CSNRT, corrected sinus node recovery time; EP, electrophysiological; fSND, functional sinus node dysfunction; fAVB, functional atrioventricular block; HR: heart rate; bpm: beats per minute; HV, His-ventricle interval; PW, Wenckebach period.

Overall, the predominant vagal phenotypes were isolated fSND in 53/123 patients (43.1%), dual presentations in 58/123 patients (47.2%), and isolated fAVB in 12/123 patients (9.8%).

Supplementary material online, *Table S3* summarizes the baseline clinical characteristics according to the clinical phenotype. The distribution of these phenotypes did not differ significantly between the tailored and standard groups (see *Table 1*). The median syncope episodes in the 12 months preceding the procedure were 4 (IQR: 0–7), without differences between groups (*P* = 0.10). Overall, 92 patients (74.8%) completed HUTT as part of their pre-procedural evaluation. Cardio-inhibitory type 2B (50.0%) was the most frequent response followed by cardio-inhibitory type 2A (19.6%), with no significant differences between groups (*P* = 0.26). Of note, HUTT was systematically performed per protocol, even in patients who already had a clear indication for CNA. Seven patients exhibited a vasodepressor response during HUTT, which does not constitute an indication for CNA; these patients had previously documented vagally induced symptomatic bradycardia identified through other diagnostic modalities (see Supplementary material online, *Table S5*). Preprocedural EP study excluded intrinsic SND or conduction system disease in all patients, showing similar parameters in the two groups. No differences were found in the atropine response between groups, as *Table 1* shows. Diagnostic tools leading to the final phenotype classification were similar between the tailored and standard groups (see Supplementary material online, *Table S4*). Patients with the fAVB phenotype were more frequently diagnosed based on Holter recordings.

### Cardioneuroablation acute results

Acute procedural results are summarized in *Table 2*. Mean procedure time was 77 ± 34 min with a mean 8.5 ± 5.3 min RF time. After the procedure, there was an increase in the heart rate after of 40 ± 31% with a final median heart rate of 72 (IQR: 61–80) bpm. The acute success rate was 91.1%. In the remaining cases, failure to demonstrate acute success was due to several factors. In two patients (1.6%), repetitive atrial fibrillation was induced during the electrophysiological study, which precluded atropine testing. In two other patients (1.6%), ablation of specific GPs was incomplete due to phrenic nerve capture—one during SPSGP ablation and the other during Ao–SVC GP ablation—limiting the procedural endpoint to partial heart rate response. Additionally, seven patients (5.7%) showed more than 25% heart rate increase after atropine test. Only one procedural complication occurred: a symptomatic pericardial effusion requiring next-day drainage in the standard group (1.4%).

**Table 2:** Procedural characteristics and outcomes by treatment group

	Standard approach	Tailored approach	*P*-value	Total population
**Procedural metrics**				
Bi-atrial ablation	69 (100)	34(63.0)	0.001	103 (83.7)
LA-only ablation	0 (0)	20 (37.0)	0.001	20 (16.3)
CNA procedure time, min	85.1 (35.9)	63.4 (25.1)	0.001	76.8 (33.7)
RF application time, min	10.4 (4.5)	5.4 (5.2)	<0.001	8.5 (5.3)
RF num. applications	25 [14–32]	14 [6.5–18.8]	<0.001	18 [9.5–28.5]
Fluoroscopy time, sec	158.9 (132.9)	177.7 (108.4)	0.51	165.1 (125.1)
**Electrophysiological parameters**				
Heart rate increase after CNA, %	39.9 (31.4)	39.7 (30.7)	0.96	39.8 (30.9)
Post-procedural basal HR, bpm	71.92 (15.38)	73.39 (16.55)	0.63	72.56 (15.84)
PR interval reduction, ms	8.83 (64.53)	1.12 (18.81)	0.34	4.90 (51.65)
Wenckebach point change, ms	51.9 (135.8)	37.4 (136.5)	0.60	46.40 (135.57)
**Procedural outcomes**				
Acute CNA success, *n* (%)	62 (90.4)	50 (92.6)	0.98	112 (91.1)
Complications, *n* (%)	1 (1,4)^a^	0 (0.0)	—	1 (0,08)

Values are presented as median [interquartile range], mean (standard deviation), or *n* (%).

bpm, beats per minute; CNA, cardioneuroablation; HR, heart rate; LA, left atrium; RF, radiofrequency.

^a^One case of symptomatic pericardial effusion requiring next-day drainage.

### Tailored approach

Whereas in the standard group all GPs were targeted for ablation, in the tailored approach GPs targeting ablation and sequence was based on the patient's vagal phenotype, as *Figure 1* shows. *Table 3* shows the GP that were finally targeted in this group. By mean, in the tailored group, 1.6 ± 0.7 GP were targeted per patient (1.1 ± 0.3 in patients with fSND, 1.3 ± 0.5 in patients with fAVB phenotype and 2.1 ± 0.5 in patients with a dual phenotype). In the 22 patients with a fSND only phenotype, ablation of the SPSGP from the LA alone achieved effective denervation in 9/22 patients (40.9%), whereas 13/22 patients (59.1%) required subsequent contralateral SPSGP and/or AO-SCV GP ablation from the RA to obtain acute procedural success. Among the four patients with an AVB-only phenotype (*n* = 4), ablation was limited to the left-sided IPSGP ablation in two out of four cases (50.0%), whereas the remaining two cases required additional right-sided IPSGP ablation to achieve adequate AV nodal modulation. *Table 3* shows the GP targeted in the remaining 28 patients presenting a dual phenotype. Of note, in one patient, we decided to perform ablation only from the RA since the left SPSGP was located anatomically distant from the left atrium, based on our preprocedural CT analysis. ECVS identified previously unrecognized dual phenotype in 8/54 tailored patients (15.1%), six patients initially classified as isolated fSND and two patients initially classified as isolated fAVB.

**Table 3:** Targeted ganglionated Plexus according to patient clinical phenotype

Phenotype	*n*	LA SPSGP	RA SPSGP	LA IPSGP	RA IPSGP	Ao–SVC GP
**fSND only**	22	22 (100%)	13 (59.1%)	—	—	2 (9.1%)
**fAVB only**	4	—	—	4 (100%)	2 (50.0%)	—
**Dual**	28	27 (96.4%)	19 (67.9%)	25 (89.3%)	14 (50.0%)	5 (17.9%)

Values are presented as percentages (*n*).

fAVB, functional atrioventricular block; fSND, functional sinus node dysfunction; GP, ganglionated plexus; IPSGP, inferior para-septal GP; LA, left atrium; Ao-SVC, aorta-superior vena cava; RA, right atrium; SPSGP, superior para-septal GP.

### Standard vs. tailored approach

When acute results were compared between groups, total procedure time was shorter in the tailored group (63 ± 25 min) compared to the standard group (85 ± 36 min; *P* = 0.001). The total RF application time was significantly reduced in the tailored group compared to the standard group (5.4 ± 5.2 vs. 10.4 ± 4.5 min, respectively; *P* < 0.001). Median RF applications were 25 (IQR: 14–32) in the standard group vs. 14(6.5–19) in the tailored group (*P* < 0.001). Fluoroscopy time was comparable in both groups (standard group 159 ± 133 s vs. tailored group 178 ± 108 s, *P* = 0.51). Acute CNA success did not differ between groups [62 patients (90%) in the standard group and 50 patients (93%) in the tailored group (*P* = 0.98)], neither heart rate increases after CNA [40 ± 31% in the standard vs. 40 ± 31% in the tailored group, *P* = 0.96).

### Follow-up and long-term clinical outcomes

**Figure 2:**
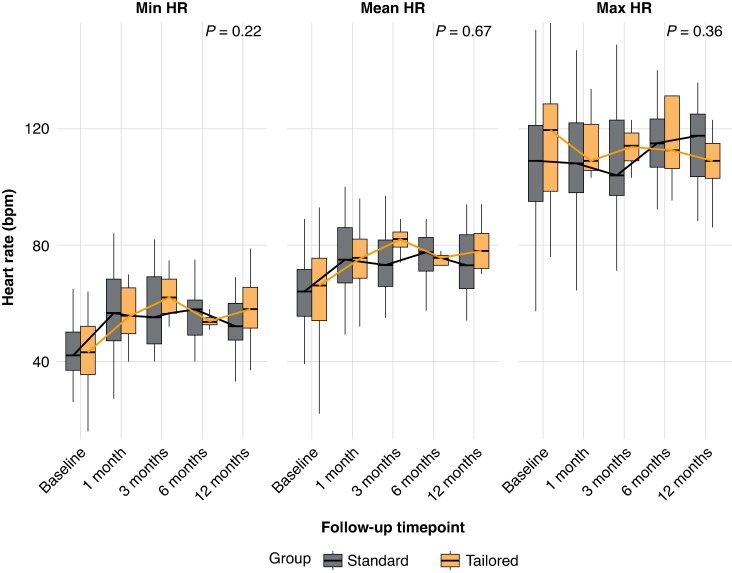
Distribution of HUTT responses before and after CNA in standard and tailored groups. The bar plot illustrates the proportional distribution of HUTT responses before and after CNA in Standard and Tailored groups. Post-CNA, both groups showed a significant increase in negative response.

Post-procedural HUTT results demonstrated significant improvement, with no significant differences in the responses in both groups (*Figure 2*). After CNA, HUTT negativization rates were comparable between the tailored approach and standard approach (63.0% vs. 53.3%, respectively, *P* = 0.359). Serial Holter monitoring during follow-up confirmed significant and persistent heart rate changes following CNA, without differences between groups. At 1-month follow-up, median heart rate increased from 65 bpm (IQR: 55–73) pre-procedure to 76 bpm (IQR: 69–83) post-procedure (*P* < 0.001), while minimum heart rate showed substantial improvement from 42 bpm (IQR: 35–50) to 56 bpm (IQR: 48–65) post-procedure (*P* < 0.001). Maximum heart rate remained essentially unchanged [pre: 110 bpm (IQR: 95–126) vs. post: 112 bpm (IQR: 102–122), *P* = 0.36].

**Figure 3:**
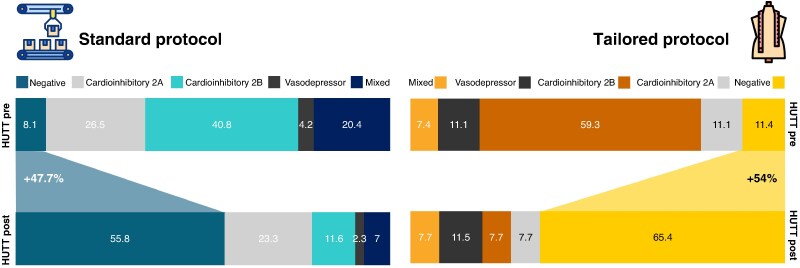
Heart rate at serial holter ECG extended serial holter monitoring demonstrated sustained HR changes across all follow-up timepoints. Both the tailored and standard CNA approaches produced comparable HR increases, with no significant differences between groups in median HR (*P* = 0.67), minimum HR (*P* = 0.22), or maximum HR (*P* = 0.36). HUTT, head-up tilt test.

Extended serial Holter monitoring revealed sustained HR changes maintained across all measured timepoints throughout the follow-up period (see *Figure 3*). Both treatment approaches produced comparable heart rate changes, with no statistically significant differences between groups in median heart rate increases (*P* = 0.67), minimum heart rate increases (*P* = 0.22), or maximum heart rate changes (*P* = 0.36), as *Figure 3* shows. HR, heart rate.

**Figure 4:**
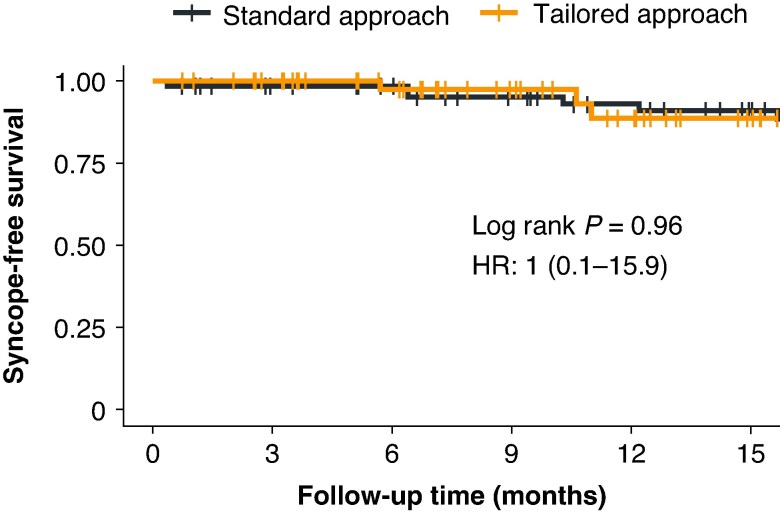
Kaplan–meier analysis of syncope-free survival: standard vs. tailored approaches. The Kaplan–Meier survival analysis compares syncope-free survival rates between patients treated with the standard approach and those treated with a tailored approach over a follow-up period. The analysis indicates no statistically significant difference between the two groups, as evidenced by a log-rank *P*-value of 0.96. Additionally, the hazard ratio of 1.0 (95% CI: 0.1–15.9) highlights equivalent outcomes between the standard and tailored approaches in preventing syncope recurrence. HR, hazard ratio.

During a median follow-up of 15.9 months (IQR: 9.8, 24.6), both cardioneuroablation approaches demonstrated similar outcomes in preventing syncope recurrence. The Kaplan–Meier survival analysis demonstrated comparable syncope-free survival between the standard and tailored approach groups over follow-up (*Figure 4*). The log-rank test revealed no statistically significant difference between the groups (*P* = 0.96). The hazard ratio of 1.0 (95% CI: 0.06–15.92) further confirmed the equivalent effectiveness of both approaches in preventing syncope recurrence. Symptoms recurrence was observed in eight patients (11.5%) in the standard group and four patients (7.4%) in the tailored group (*P* = 0.76).

Symptomatic inappropriate sinus tachycardia was observed in 10 patients (8.1% of total cohort), with similar distribution between groups [6/69 (8.6%) in the standard approach group vs. 4/54 (7.4%) in the tailored approach group, *P* = 0.79]. Treatment strategies included ivabradine (*n* = 6) and low-dose beta-blockers (*n* = 4), with both regimens showing good tolerability and symptomatic improvement. No other significant complications were observed.

## Discussion

The main findings of this study can be summarized as follows: (i) patients considered for cardioneuroablation can be effectively classified according to their predominant clinical phenotype, allowing for a more individualized treatment strategy; (ii) an ablation approach tailored to the clinical phenotype significantly simplifies the procedure by requiring ablation of, on average, only 1.6 GPs; (iii) This phenotype-guided strategy results in a significant reduction in both total procedure time and cumulative RF energy delivery, without compromising the acute procedural success; and (iv) the tailored approach demonstrates similar outcomes in terms of recurrence-free survival when compared with the standard, more extensive technique that involves unselective ablation of all GPs.

### Physiological rationale behind the tailored strategy

Functionally, the right vagus nerve predominantly innervates the sinoatrial node, while the left vagus nerve primarily influences the AV node.^7^ Anatomical studies have shown that the SPSGP serves as the final common pathway for right vagal innervation of the sinoatrial node, whereas the IPSGP represents the corresponding structure for left vagal innervation of the AV node.^9^ Consistent with this anatomy, different physiological responses have been reported following ablation of distinct GPs. Specifically, ablation of the SPSGP and Ao-SVC GP is associated with significant increases in heart rate.^12^ In contrast, improvement in AV nodal conduction is predominantly observed after ablation IPSGP.^10,13^ Interestingly, ablation of other GPs—such as the LSGP, LIGP, and RIGP—has been associated with little or no change in heart rate,^11^ underscoring the importance of targeted selection. Therefore, a personalized ablation strategy tailored to the predominant clinical manifestation of the individual patient could be the best approach. In line with this, a recent EHRA document recommend to modulate the ablation based on the patient symptoms.^4^ Although selecting the GP to target based on the predominant clinical manifestation has been previously described,^14, 15^ the present study offers the first description of a systematic tailored approach individualized to each patient’s specific clinical profile. The present data support the rationale and potential clinical benefit of this individualized strategy. The strategy of mainly targeting the SPSGP for patients with predominant sinus node bradycardia whereas the IPSGP in case on AV node affectation provide good results in acute and long term. Regarding other GPs, adjunctive ablation of the Ao-SVC GP was required in one out of six of patients to achieve a full vagal denervation, especially in the sinus-node bradycardia and in the dual phenotype. Targeting of Ao-SVC GP was also useful in cases in which phrenic nerve capture preclude SPSGP ablation. In contrast, none of the atrioventricular-block–only patients needed Ao–SVC GP after inferior plexi had been ablated.

### Comparison with other ablative approaches

The optimal ablation strategy for CNA remains to be defined. Since both the SPSGP and IPSGP are accessible from the RA, a strategy exclusively targeting these GPs from the RA has been proposed.^16^ This approach offers the advantage of simplifying the procedure and avoiding the risks associated with transseptal puncture. However, a recent meta-analysis showed that RA-only ablation is associated with a lower rate of freedom from syncope.^17^ In contrast, biatrial ablation has been associated with a 92% freedom from syncope and was the strategy used in the only randomized controlled trial to date.^6, 17^ Francia et al. recently reported that, in the context of biatrial ablation, most of the clinical benefit is achieved during LA ablation.^18^ They observed that acute improvement in AV node conduction was achieved almost exclusively from the LA, and that initiating CNA in the LA was associated with better outcomes. Similarly, a recent randomized study report that acute total vagal denervation of the AV node is obtained significantly more frequently using the LA approach than the RA approach.^19^ These findings are consistent with our results. In the present study, AV conduction improvement was obtained without requiring right-sided IPSGP ablation in half of the cases, supporting the predominant role of left-sided IPSGP clusters in modulating atrioventricular autonomic function. Similarly, targeting the SPSGP from the LA alone was sufficient to achieve the desired sinus node modification effect in 40% of cases.

While the role of ablating the IPS- and SPS-GPs appears well established, the role of the remaining GPs is more controversial. Minguito-Carazo and colleagues recently reported the results of an observational study including 58 patients in which they compared a simplified approach—systematically targeting three GPs irrespectively of the patient’s phenotype—with a comprehensive ‘all-GPs’ approach.^20^ The simplified strategy achieved similar autonomic denervation and clinical outcomes to the extended ablation, while significantly reducing total procedure time. The findings of the present study are consistent with these results, emphasizing the importance of considering the patient’s clinical phenotype to further personalize and streamline the procedure.

Finally, a personalized strategy encompassed not only the ablation approach but also the definition of acute procedural end-points tailored to the predominant clinical presentation. For instance, in patients with fSND and persistent functional bradycardia, the procedural goal included to achieve at least 70% of the heart rate increase previously observed during the preprocedural atropine test. This criterion was not applied to other subgroups, in order to prevent excessive post-ablation tachycardia. Adoption of these individualized end-points was associated with a minimal complication rate, including a low need for ivabradine therapy during follow-up.

### Limitations

Although baseline characteristics were well-matched between groups, the non-randomized nature of the study introduces potential selection bias. Extracardiac vagal stimulation for intraprocedural success verification was not used in all patients. Since the standard and tailored approaches were implemented sequentially, we cannot exclude that increasing operator experience over time could have played a role in the outcomes reported in this study. The subgroup of patients with the fAVB clinical phenotype was small, which precluded a meaningful analysis of the potential beneficial effects of the tailored approach on post-CNA inappropriate sinus tachycardia occurrence in this specific subgroup. Consequently, the similar inappropriate sinus tachycardia rates observed between the standard and tailored approaches (7.4% vs. 8.6%; *P* = 0.79) should be interpreted with caution, as they primarily reflect outcomes in patients with fSND and dual phenotypes. Further studies specifically focused on the fAVB population are warranted to determine whether avoiding superior GP ablation in these patients may reduce post-CNA inappropriate sinus tachycardia.

## Conclusions

Our study demonstrates that a tailored CNA approach, targeting only pathophysiologically relevant GPs based on individual clinical presentation, achieves comparable clinical outcomes to the standard comprehensive strategy while significantly reducing procedure time and RF application duration. The similar acute success rates and recurrence rates between groups suggest that selective GP ablation may offer a more efficient treatment option without compromising therapeutic efficacy. These findings support a shift towards more personalized CNA strategies; however, further prospective studies with longer follow-up periods are warranted to validate these results.

## Supplementary Material

euaf320_Supplementary_Data

## Data Availability

The data that support the findings of this study are available from the corresponding author, upon reasonable request.
