# Supplementary material for: A tailored approach to cardioneuroablation for reflex syncope and functional bradycardia: results from the ELEGANCE multicentre study
Source: Europace. 2025 Dec 30;28(1):euaf320. doi: 10.1093/europace/euaf320 (PMC12849814; doi:10.1093/europace/euaf320)
Supplement: euaf320_Supplementary_Data [file euaf320_supplementary_data.docx]

A Tailored Approach to Cardioneuroablation for Reflex Syncope and Functional Bradycardia. Results from the ELEGANCE multicenter study

Carlo Gigante^1,2^*, Diego Penela^3^*, Daniel Viveros^1,2^, Giulio Falasconi^1,2^, Lucio Teresi^1,2^, Alessia Chiara Latini^3,4^, David Soto-Iglesias^1^, Paula Franco-Ocaña^1^, Pietro Francia^5,6^, José Alderete^1,2^, Dario Turturiello^1,2^, Aldo Francisco Bellido^1,7^, Fatima Zaraket^1^, Chiara Valeriano^3^, Roberta Mea^1,8^, Bruno Tonello^1^, Lautaro Sanchez-Mollá^1^, Carmine De Lucia^1^, Maria Matiello^9^, Juan Fernández-Armenta^10^, Rodolfo San Antonio^11^, Andrea Saglietto^12,13^, José-Tomás Ortiz-Pérez^1,2^, Julio Marti-Almor^1^, Antonio Berruezo^1^

* The present Authors equally contributed to this article

1 Arrhythmia Department, Heart Institute, Teknon Medical Center, c. Villana, 12, Barcelona 08022, Spain

2 Facultat de Medicina I Ciències de la Salut, Universitat de Barcelona (UB), c. Casanova, 143, Barcelona 08036, Spain

3 Arrhythmia Department, Humanitas Research Hospital IRCCS, Rozzano, Milan, Italy.

4 Department of Biomedical Sciences, Humanitas University, Pieve Emanuele-Milan, Italy.

5 Cardio Cardiology Department, St. Andrea Hospital, Rome, Italy

6 Department of Clinical and Molecular Medicine, Sapienza University, Rome, Italy

7 Arrhythmia Department, Hospital El Pilar, Barcelona, Spain.

8 Department of Biomedical and Clinical Sciences, University of Milan, Milan, Italy

9 Arrhythmia Department, Hospital General de Catalunya, Barcelona, Spain

10 Arrhythmia Department, Puerta del Mar University Hospital, Cádiz, Spain

11 Cardiology Department, Bellvitge University Hospital, BIO-HEART Cardiovascular Research Group (IDIBELL), l'Hospitalet de Llobregat, Barcelona, Spain

12 Division of Cardiology, Cardiovascular and Thoracic Department, Città della Salute e della Scienza Hospital, Turin, Italy.

13 Department of Medical Sciences, University of Turin, Turin, Italy.

Address for correspondence

Antonio Berruezo, MD, PhD.

Arrhythmia Department, Teknon Heart Institute

Teknon Medical Center, Barcelona, Spain

Tel: +34 93 290 6200; fax: +34 932 11 26 90

E-mail: [antonio.berruezo@quironsalud.es](mailto:antonio.berruezo@quironsalud.es)

**Extracardiac Vagal Stimulation Protocol**

Extracardiac vagal stimulation (ECVS) was employed when available to assess the completeness of cardiac vagal denervation following cardioneuroablation procedures. The technique was implemented according to the standardized protocol established by Pachón et al., utilizing a dedicated vagal stimulator (Pachón & Pachón, São Paulo, Brazil) configured for direct current stimulation delivery. Stimulation parameters were standardized with square wave pulses of 50 μs duration, delivered at a frequency of 30 Hz with amplitude ranging from 10 to 70 V, individually adjusted according to patient body weight (0.5 to 1.0 V/kg). The extremely short pulse duration combined with current limitations was specifically designed to prevent inadvertent tissue lesions while maintaining effective neural stimulation. A programmable timer function enabled the delivery of pulse trains with predefined durations, typically ranging from 8 to 12 seconds.The stimulation was achieved through creation of an endovascular electrical field within the internal jugular vein, utilizing either the distal and third pole of the ablation catheter (temporarily detached from the radiofrequency generator) or a standard diagnostic tetrapolar catheter. This approach avoided direct contact with the vagus nerve, with energy delivery titrated according to the variable anatomical distance between the vagus nerve and the intravascular catheter. The optimal stimulation site was systematically identified within the range extending from the lower boundary of the orbit to the base of the wisdom tooth.All procedures were performed under general anesthesia with bispectral index (BIS) monitoring maintained between 40 and 50 to ensure consistent autonomic tone across all evaluations while preventing significant autonomic depression and maintaining patient safety and comfort. Monolateral or bilateral vagal stimulation was performed systematically before and after ablation using identical parameters and positioning.The procedural protocol involved systematic exploration to identify the position yielding maximum vagal response, characterized by the induction of sudden cardioinhibition manifesting as sinus arrest, bradycardia, and/or atrioventricular block. Following initial assessment, ECVS was repeated under atrial pacing at rates 10 to 20 beats per minute higher than the baseline sinus rate to induce transient complete atrioventricular block. Subsequently, identical vagal stimulation was performed in the contralateral internal jugular vein. The anatomical positions demonstrating optimal vagal responses were systematically marked using fluoroscopic guidance, with 8 to 12 seconds of stimulation delivered and recorded at each optimal site.Post-ablation ECVS was conducted using identical stimulation parameters and positioning to dynamically assess denervation progression throughout the procedure and to confirm complete vagal denervation as the definitive procedural endpoint. Complete vagal denervation was defined as the absence of cardioinhibitory response following standardized extracardiac vagal stimulation, establishing successful cardiac autonomic modulation.

**Supplementary Table 1. Criteria for Acute Procedural Success According to Patient Phenotype**

| **Clinical Phenotype** | **Primary Intraprocedural Endpoint** | **Supportive Criteria** | **Subset with ECVS availability** |
| --- | --- | --- | --- |
| **Functional Sinus Node Dysfunction (fSND)** | HR increase >25% from baseline | - Negative CSM after ablation  - <25% HR increase after atropine | Abolition of cardioinhibitory response to ECVS |
| **fSND presenting with Persistent Functional Bradycardia** | HR increase ≥70% of HR response to atropine achieved in the baseline EP study | - Negative CSM after ablation  - <25% HR increase after atropine |  |
| **Functional Atrioventricular Block (fAVB)** | Resolution of AV block and/or ≥25% PR interval reduction | - Negative CSM after ablation  - No further PR shortening after atropine | Abolition of cardioinhibitory response to ECVS |
| **Dual Phenotype (fSND + fAVB)** | HR increase >25% from baseline  and  Resolution of AV block and/or ≥25% PR interval reduction | Combination of all of above endpoints | Abolition of cardioinhibitory response to ECVS |

*HR = heart rate; CSM = carotid sinus massage; AVB = atrioventricular block; EP = electrophysiology; ECVS = extracardiac vagal stimulation. fSND = Functional Sinus Node Dysfunction; fAVB = Functional Atrioventricular Block.*

**Personalized GP ablation with local LAWT-guided ablation target.**

Ablation targets were titrated according to the local thickness of the 3D left atrial wall thickness (LAWT) map; briefly, the following Ablation Index (AI) from Biosense Webster targets were used: 400 for LAWT <1 mm (red zones), 450 for LAWT ≥1 mm and < 2 mm (yellow zones), 500 for LAWT ≥2 mm and < 3 mm (green zones), 550 for LAWT ≥3 mm and < 4 mm (blue zones), and 600 for LAWT ≥4 mm (purple zones).

AI targets were adjusted based on the local LAWT with the aim of achieving transmural lesion formation and deeper epicardial reach. In contrast to prior protocols, our strategy employed AI values approximately 100 units higher for each LAWT range to enhance lesion depth and optimize parasympathetic denervation. The corresponding Lesion Size Index (LSI) from Abbott are also provided below for reproducibility across platforms.

***Supplementary Table 2****. Personalized GP ablation by local Left Atrial Wall Thickness (LAWT). Note that these values are based on the LAWT-guided ablation index required to achieve a transmural lesion, derived from AF ablation protocols, and increased by 100 units to reach the epicardially located GPs.*

| **Left Atrial Wall Thickness (LAWT)** | **Color Code** | **Ablation Index (AI)** | **Lesion Size Index (LSI)** | **RF Power** |
| --- | --- | --- | --- | --- |
| <1 mm | Red | 400 | 4.0 | 45 W |
| 1–2 mm | Yellow | 450 | 4.5 | 45 W |
| 2–3 mm | Green | 500 | 5.0 | 45 W |
| 3–4 mm | Blue | 550 | 5.5 | 45 W |
| >4 mm | Purple | 600 | 6.0 | 45 W |


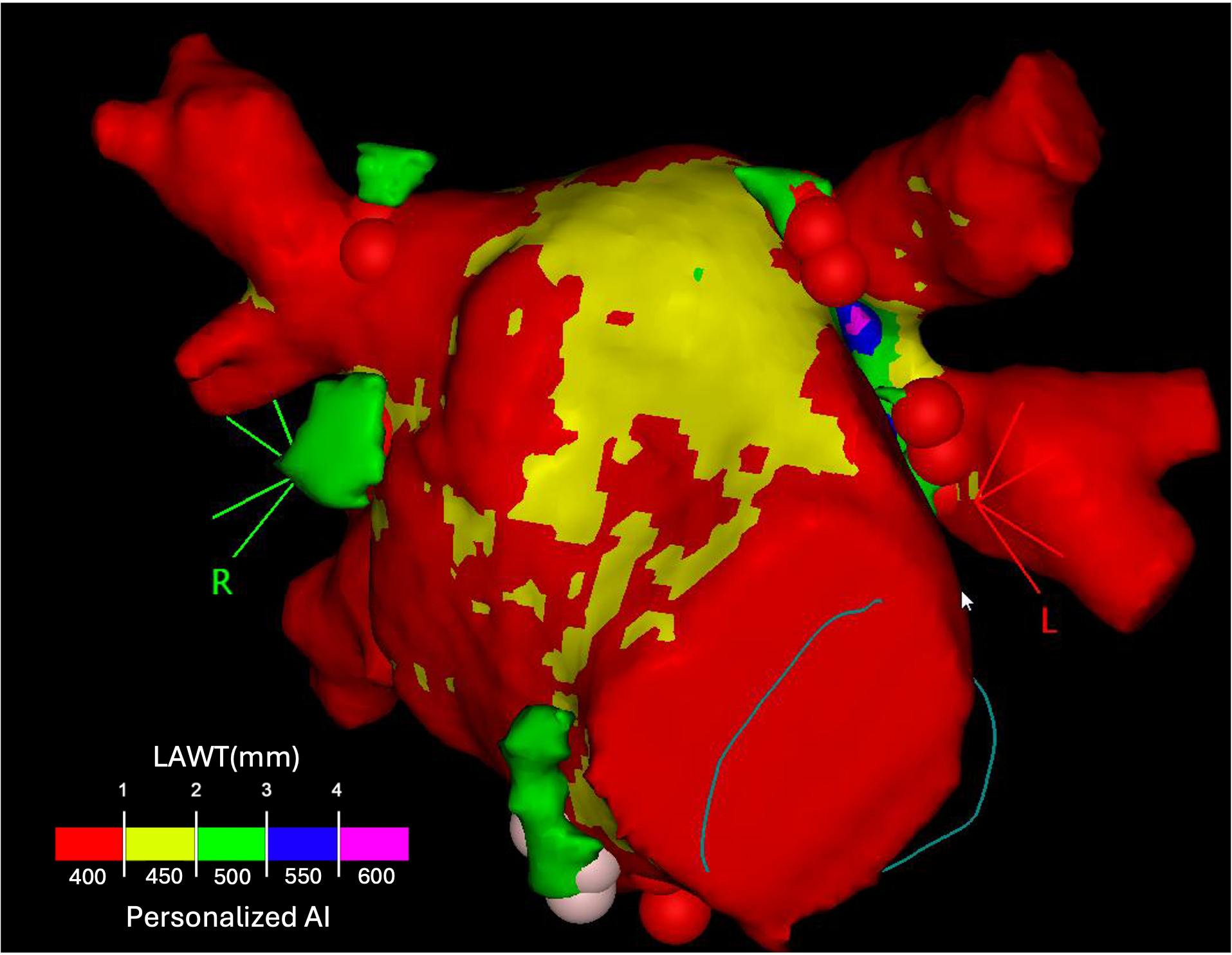


**Supplementary Figure 1** Personalized GP ablation by local Left Atrial Wall Thickness (LAWT)

At left, an example of a LAWT-guided CNA. The LAWT map is color-coded in the following manner: red for LAWT < 1 mm, yellow for LAWT between 1 and 2 mm, green for LAWT between 2 and 3 mm, blue for LAWT between 3 and 4 mm, and purple for LAWT ≥ 4 mm. Al targets were adjusted according to the underlying LAWT in a point-by-point fashion.

**Supplemental Table 3: Clinical and Demographic Characteristics by Phenotype (All Patients, n=123)**

| **Variable** | **fSND (n=53)** | **fAVB (n=12)** | **Dual (n=58)** | **Total (n=123)** | **p-value** |
| --- | --- | --- | --- | --- | --- |
| **Female sex** | 23 (43.4%) | 5 (41.7%) | 22 (37.9%) | 50 (40.7%) | 0.840 |
| **Age (years)** | 56.5 [40.8, 67.0] | 56.5 [41.8, 66.5] | 46.0 [34.0, 58.8] | 50.0 [36.0, 63.0] | 0.053 |
| **Number of Syncopes** | 3.0 [0.0, 7.0] | 1.0 [0.0, 3.8] | 3.0 [0.8, 7.0] | 3.0 [0.0, 6.0] | 0.259 |
| **Holter mean HR (bpm)** | 63.5 [54.0, 74.5] | 62.0 [53.0, 80.0] | 65.0 [57.5, 70.5] | 65.0 [55.0, 73.0] | 0.962 |
| **Holter minimum HR (bpm)** | 42.0 [35.8, 49.2] | 37.0 [29.0, 44.0] | 43.0 [36.0, 52.0] | 42.0 [35.0, 50.0] | 0.240 |
| **Holter maximum HR (bpm)** | 110.0 [94.2, 122.5] | 106.0 [101.0, 144.0] | 110.0 [95.0, 127.0] | 110.0 [95.0, 126.2] | 0.626 |
| **Hypertension** | 9 (17.0%) | 2 (16.7%) | 8 (13.8%) | 19 (15.4%) | 0.891 |
| **Dyslipidemia** | 5 (9.4%) | 3 (25.0%) | 4 (6.9%) | 12 (9.8%) | 0.156 |
| **Diabetes mellitus** | 1 (1.9%) | 0 (0.0%) | 0 (0.0%) | 1 (0.8%) | 0.514 |
| **Smoking** | 2 (3.8%) | 0 (0.0%) | 3 (5.2%) | 5 (4.1%) | 0.704 |
| **Sleep apnea** | 2 (3.8%) | 0 (0.0%) | 2 (3.4%) | 4 (3.3%) | 0.796 |
| **Atrial fibrillation** | 9 (17.0%) | 2 (16.7%) | 2 (3.4%) | 13 (10.6%) | 0.053 |

*fSND = Functional Sinus Node Dysfunction; fAVB = Functional Atrioventricular Block.*

**Supplementary Table 4A – This table shows the diagnostic tool that contributes to identify the vagal phenotype based on Treatment Group**

| **Diagnostic Method (N patients tested)** | **Standard (n=69)** | **Tailored (n=54)** | **p-value** |
| --- | --- | --- | --- |
| **Holter monitoring (n=123)** | 43.5% (30/69) | 29.6% (16/54) | 0.165 |
| **Implantable loop recorder (n=7)** | 75.0% (3/4) | 3/3 (100.0%) | 0.444 |
| **Tilt test (n=92)** | 88.5% (46/52) | 80.0% (32/40) | 0.233 |
| **Electrophysiologic study (n=123)** | 34.8% (24/69) | 29.6%(16/54) | 0.681 |

**Supplementary Table 4B – This table shows the Diagnostic Tool that contributes to clarify the patient’s phenotype**

| **Diagnostic Method**  **(N patients tested)** | **fSND (n=53)** | **fAVB (n=12)** | **Dual (n=58)** | **p-value** |
| --- | --- | --- | --- | --- |
| **Holter monitoring (123)** | 17%(9/53) | 83%(10/12) | 46.6%(27/58) | 0.0004 |
| **Implantable loop recorder (7)** | 100% (2/2) | 100% (1/1) | 75%(3/4) | 0.478 |
| **Tilt test (92)** | 83%(29/35) | 57% (4/7) | 90%(45/50) | 0.069 |
| **Electrophysiologic study (123)** | 24.5%(13/53) | 50.0%(6/12) | 36.2%(21/58) | 0.168 |

*fSND = Functional Sinus Node Dysfunction; fAVB = Functional Atrioventricular Block.*

**Supplementary Table 5.** Clinical characteristics of patients with vasodepressor HUT response (n=7)

| **Patient** | **Age** | **Sex** | **Syncope** | **HUT Response** | **Primary CNA Indication** | **Diagnostic Modality** |
| --- | --- | --- | --- | --- | --- | --- |
| 1 | 57 | F | No | Vasodepressor | Symptomatic sinus bradycardia | Holter monitoring |
| 2 | 57 | F | Yes | Vasodepressor | Functional complete AV block | Implantable loop recorder |
| 3 | 67 | M | Yes | Vasodepressor | Cardioinhibitory CSH | Carotid sinus massage |
| 4 | 50 | M | Yes | Vasodepressor | Cardioinhibitory CSH | Carotid sinus massage |
| 5 | 32 | M | Yes | Vasodepressor | Symptomatic sinus bradycardia | Holter monitoring |
| 6 | 43 | M | No | Vasodepressor | Symptomatic sinus bradycardia | Holter monitoring |
| 7 | 35 | F | Yes | Vasodepressor | Functional complete AV block | Holter monitoring |

*Abbreviations: HUT = head-up tilt test; CNA = cardioneuroablation; AV = atrioventricular; CSH = carotid sinus hypersensitivity.*
